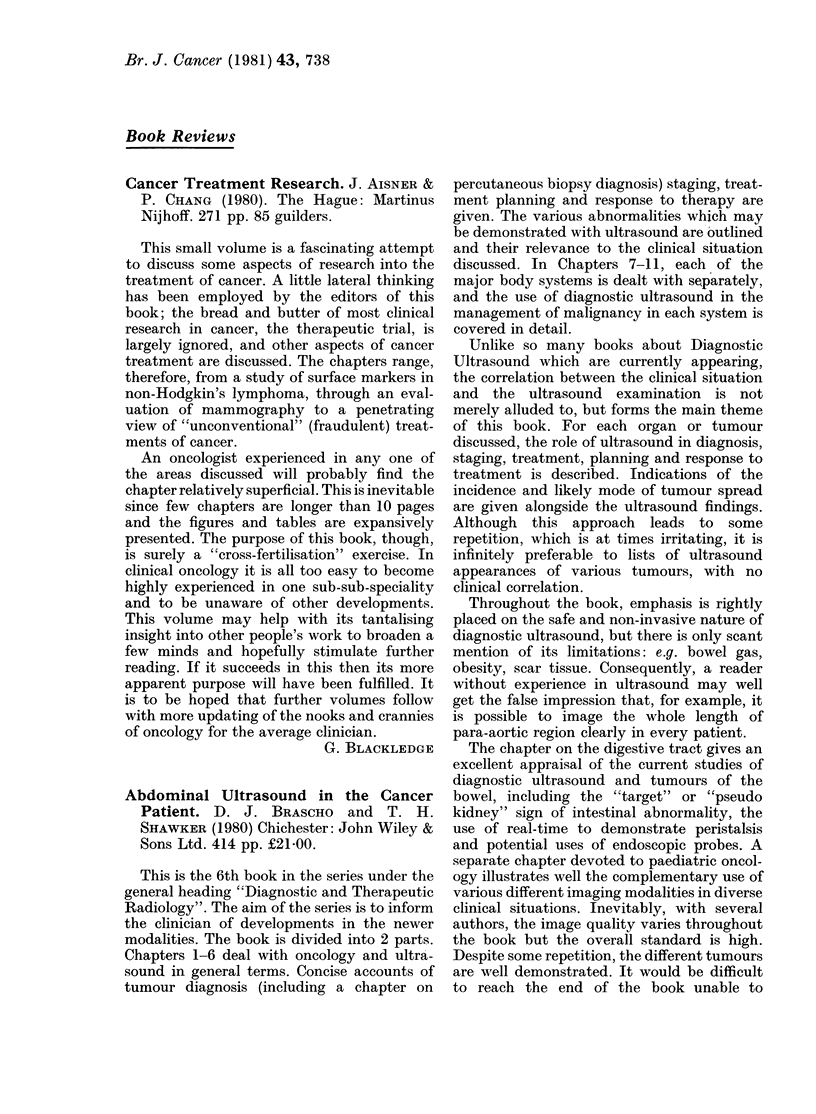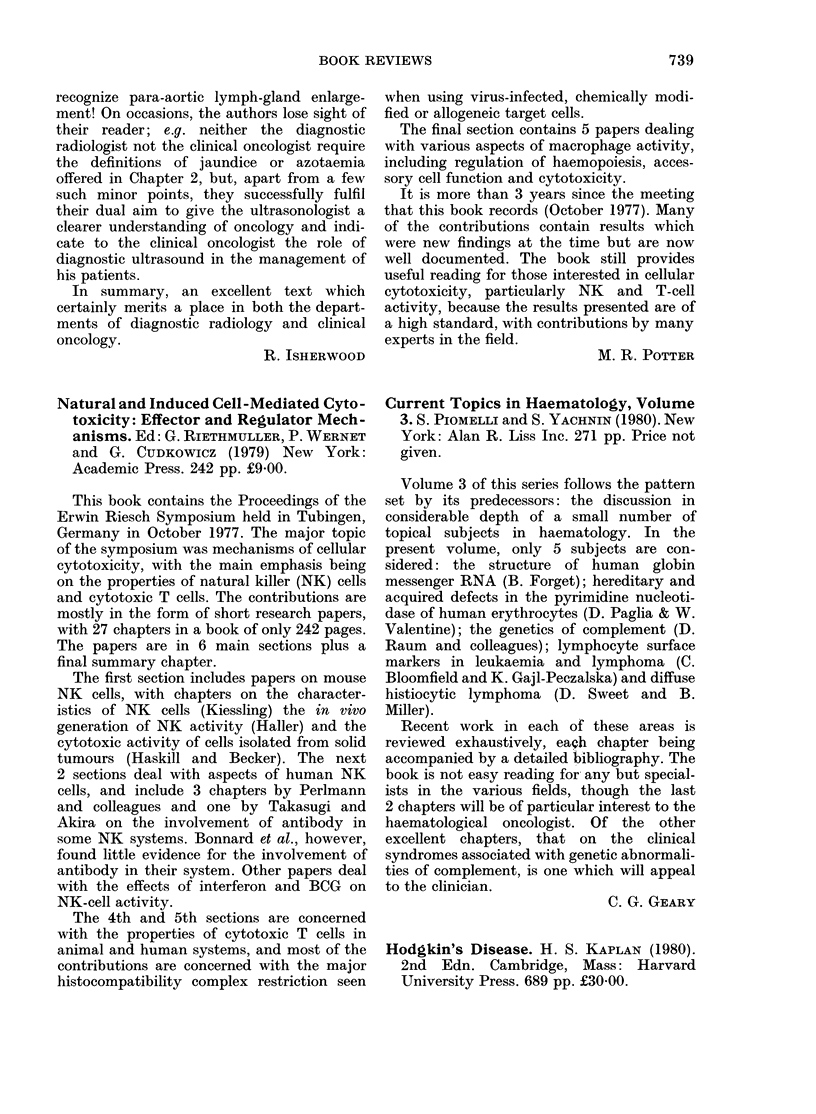# Abdominal Ultrasound in the Cancer Patient

**Published:** 1981-05

**Authors:** R. Isherwood


**Abdominal Ultrasound in the Cancer Patient.** D. J. Brascho and T. H. Shawker (1980) Chichester: John Wiley & Sons Ltd. 414 pp. £21·00.

This is the 6th book in the series under the general heading "Diagnostic and Therapeutic Radiology". The aim of the series is to inform the clinician of developments in the newer modalities. The book is divided into 2 parts. Chapters 1–6 deal with oncology and ultrasound in general terms. Concise accounts of tumour diagnosis (including a chapter on percutaneous biopsy diagnosis) staging, treatment planning and response to therapy are given. The various abnormalities which may be demonstrated with ultrasound are outlined and their relevance to the clinical situation discussed. In Chapters 7–11, each of the major body systems is dealt with separately, and the use of diagnostic ultrasound in the management of malignancy in each system is covered in detail.

Unlike so many books about Diagnostic Ultrasound which are currently appearing, the correlation between the clinical situation and the ultrasound examination is not merely alluded to, but forms the main theme of this book. For each organ or tumour discussed, the role of ultrasound in diagnosis, staging, treatment, planning and response to treatment is described. Indications of the incidence and likely mode of tumour spread are given alongside the ultrasound findings. Although this approach leads to some repetition, which is at times irritating, it is infinitely preferable to lists of ultrasound appearances of various tumours, with no clinical correlation.

Throughout the book, emphasis is rightly placed on the safe and non-invasive nature of diagnostic ultrasound, but there is only scant mention of its limitations: *e.g.* bowel gas, obesity, scar tissue. Consequently, a reader without experience in ultrasound may well get the false impression that, for example, it is possible to image the whole length of para-aortic region clearly in every patient.

The chapter on the digestive tract gives an excellent appraisal of the current studies of diagnostic ultrasound and tumours of the bowel, including the "target" or "pseudo kidney" sign of intestinal abnormality, the use of real-time to demonstrate peristalsis and potential uses of endoscopic probes. A separate chapter devoted to paediatric oncology illustrates well the complementary use of various different imaging modalities in diverse clinical situations. Inevitably, with several authors, the image quality varies throughout the book but the overall standard is high. Despite some repetition, the different tumours are well demonstrated. It would be difficult to reach the end of the book unable to recognize para-aortic lymph-gland enlargement! On occasions, the authors lose sight of their reader; *e.g.* neither the diagnostic radiologist not the clinical oncologist require the definitions of jaundice or azotaemia offered in Chapter 2, but, apart from a few such minor points, they successfully fulfil their dual aim to give the ultrasonologist a clearer understanding of oncology and indicate to the clinical oncologist the role of diagnostic ultrasound in the management of his patients.

In summary, an excellent text which certainly merits a place in both the departments of diagnostic radiology and clinical oncology.

R. ISHERWOOD